# A 3-minute test of cardiorespiratory fitness for use in primary care clinics

**DOI:** 10.1371/journal.pone.0201598

**Published:** 2018-07-30

**Authors:** Yi Guo, Jiang Bian, Qian Li, Trevor Leavitt, Eric I. Rosenberg, Thomas W. Buford, Megan D. Smith, Heather K. Vincent, François Modave

**Affiliations:** 1 Department of Health Outcomes and Biomedical Informatics, University of Florida, Gainesville, FL, United States of America; 2 Department of Orthopaedics and Rehabilitation, University of Florida, Gainesville, FL, United States of America; 3 Division of General Internal Medicine, Department of Medicine, University of Florida, Gainesville, FL, United States of America; 4 Department of Medicine, University of Alabama at Birmingham, Birmingham, AL, United States of America; University of Zurich, SWITZERLAND

## Abstract

**Background:**

Cardiorespiratory fitness (CRF) is the only major risk factor that is not routinely assessed in the clinical setting, for preventive medicine. A valid and practical CRF test is needed for use in the clinics. The objective of this study is to demonstrate the validity of a 3-minute squat test to assess CRF in primary care.

**Methods:**

A cross-sectional study in which the participants performed both the Ruffier squat test and the Balke maximal treadmill test. The study was conducted in a clinical setting from September 2016 to March 2017. We recruited a convenient sample of 40 adults between 18 and 64 years from the general U.S. population. Participants completed 30 squats in 45 seconds, paced by a metronome. Heart rate was measured at rest (P1), immediately after the test (P2), one minute after the test (P3). $\dot{V}O_{2}\max$ was measured using the Balke maximal treadmill fitness test.

**Results:**

Of the 40 participants, there were 18 men and 22 women. Mean age was 31.2 years (SD = 9.9). We found that the best $\dot{V}O_{2}\max$ predictors were HR features *P*1/*height* and (*P*2–*P*3)/*age*^3^. Our best-performing model using these two features predicted individuals’ CRF levels with an adjusted *R*^2^ of 0.637, sensitivity of 0.79, and specificity of 0.56.

**Conclusions:**

The study provided strong evidence for the validity of the squat test in the clinical setting. Further, the equation of our model along with $\dot{V}O_{2}\max$ normative tables provides an efficient and easy way to assess CRF in a primary care setting.

## Introduction

Low levels of physical activity remain a major contributor to obesity, chronic illnesses, and mortality [1]^,^[2]. Over two in three Americans are either overweight or obese, yielding an estimated US $147 billion annually in health care costs [3], while only 20% of Americans currently meet the current physical activity recommendations [4]. A direct consequence of obesity and insufficient physical activity is reduction in cardiorespiratory fitness (CRF), a strong predictor of cardiovascular events and mortality [5–10]. A meta-analysis of 33 studies inclusive of more than 100,000 participants revealed that individuals with low CRF had a 56% higher risk of cardiovascular disease mortality and a 70% higher risk of all cause-mortality compared to those with high CRF[5]. Further, strong associations exist between CRF and other health outcomes, such as depression [11]^,^[12], dementia [11–14], and cancer-related mortality [15]^,^[16]. Therefore, testing CRF is essential for preventive medicine [17,18], as exemplified by the American Heart Association (AHA) recently published policy statement highlighting the need to develop a national CRF registry [19].

Despite the clinical importance of CRF for preventive medicine, it is the only major risk factor that is not routinely assessed in the clinical setting [19]. The gold standard method for measuring CRF is the maximal oxygen uptake ($\dot{V}O_{2}\max$) test [20]. However, cost, equipment requirements, and time constraints limit its use in clinics. Other tests with moderate to good correlation with $\dot{V}O_{2}\max$ are available to assess CRF [21–25], but suffer drawbacks that make them impractical for use in primary care. For instance, the Harvard Step Test and its more recent variations [26–29] are difficult to standardize and may lead to falls. Similarly various other performance tests (e.g. walking tests, the shuttle test, the Cooper run test, and the Boucher-Léger test) cannot be implemented safely in routine clinic visits [22–25]. Thus, a simple, easy-to-administer fitness test that can be performed in outpatient settings with high patient volume is needed to measure and monitor CRF.

The Ruffier test is a 3-minute heart rate (HR)-based CRF test widely used in France [30]^,^[31]. This simple test requires participants to perform 30 squats in 45 seconds. Three measurements of HR are taken: pre-test resting HR, HR immediately after performing the squats, and recovery HR 60 seconds post-test. The three HRs are then used to compute the Ruffier-Dickson index (RDI) and Ruffier Index (RI), which can be used to classify CRF. The only research study on the validity of the Ruffier test in a general population was conducted in France [32]. To our knowledge, the validity of this test has not been studied in the U.S. population. Further, it is possible that other HR-based features may outperform the RDI for measuring CRF because the original test was developed using a different population.

In this study, we developed and validated a $\dot{V}O_{2}\max$ prediction model using data from the Ruffier test with participants from the general U.S. population. We recruited participants to sequentially perform the Ruffier test and the treadmill $\dot{V}O_{2}\max$ test. We built regression models to predict $\dot{V}O_{2}\max$ values using a combination of individuals’ characteristics, the RDI and RI, and HR features such as recovery HR from the Ruffier test. Models’ stability and performance were evaluated using adjusted *r*^2^, root-mean-squared error (RMSE), sensitivity, specificity, and Kappa. The goal of the study was to show that the Ruffier test is a valid and efficient way to assess CRF in the clinical setting.

## Methods

This study was approved by the University of Florida (UF) Institutional Review Board on 08/30/2016 (IRB2016600991). We recruited 40 participants (22 women, 18 men) between 18 and 64 years from the general North Central Florida region using flyers. We excluded individuals who: 1) were unable to walk on a treadmill continuously for at least 15 minutes, 2) had any injuries or medical conditions that prohibit exercise, 3) were taking any medicine that might affect balance (e.g, antihypertensive medication), 4) had a history of diseases that might affect balance, (e.g, stroke), 5) were pregnant, and 6) had known history of heart disease, previous heart surgeries, coronary artery vessel blockages, arrhythmias, or history of myocardial infarction.

Eligible participants were scheduled to come to the UF Sports Performance Center to complete the informed consent process and the fitness tests. Prior to testing, the study coordinator described the study protocol, consented participants, and collected baseline information from each participant, including age, sex, race, ethnicity, height, and weight. The participants were then instructed to perform the Ruffier test, in which they completed 30 squats in 45 seconds. After completing the Ruffier test and resting for 15 minutes, the participants performed the $\dot{V}O_{2}\max$ Treadmill Exercise test.

Before the Ruffier test, the research coordinator attached a 12-lead electrocardiogram (Quinton Q Stress; Cardiac Science Corporation, Bothell WA, USA) to the participant and instructed him or her to sit and rest for 5 minutes. Resting HR was collected at the end of 5 minutes (P1). The participant was then asked to complete 30 squats in 45 seconds, paced by a metronome. The squatting required moving up and down, bending the knees to a 90 degree angle, while keeping the back straight and the arms extended straight forward. HR was collected immediately after the squats (P2). Upon completion of the squats, the participant was asked to sit and recover for one minute. A third measurement of HR was obtained at one minute post-test (P3). Based on the three HR measurements, the Ruffier Index (RI) was calculated as:

$$RI=\frac{P1+P2+P3-200}{10},$$

and the Ruffier-Dickson Index (RDI) was calculated as:

$$RDI=\frac{\left(P2-70)+2\times (P3-P1\right)}{10}.$$

Using HRs measured in the Ruffier test, we have also built HR features for predicting $\dot{V}O_{2}\max$. These HR features are defined as any change in HR from one time point to another, such as the difference in HR from P2 to P3, or a HR value relative to height or age, similar to those reported by Sartor et al [32]. The Ruffier test has demonstrated validity in European populations and good test-retest reliability (intra-class coefficients of the HR features and RDI ranged from 0.66–0.86) [32].

After the squat test, the participants were asked to sit and rest for at least 15 minutes, allowing the HR to decrease to approximately the initial resting HR. We implemented a modified Balke treadmill protocol. This protocol increased cardiac workload more gradually than other protocols, thereby ensuring that individuals with of all fitness levels could participate [33].

Before the test, the study coordinator explained the entire test protocol and connected a metabolic gas analyzer (Viasys, CareFusion; Yorba Linda, CA; USA) with the participant via a rubberized facemask and a turbine. Based on the test protocol, the treadmill speed remained at 3.3 mph and the workload of the heart was increased by adding elevation. For the first minute, the incline was set at 0%. For the second minute, the incline was set at 2%. We then increased the incline by 1% per minute up to a maximal incline of 25%. If the participant achieved 25 minutes, we kept the incline at 25% and increased the speed by 0.2 mph/minute until exhaustion. During the test, we captured breath-by-breath gas exchange ventilation and respiratory rate. Rating of perceived exertion was used as a subjective measure of participant level of effort at each stage of the test using the 6–20 Borg scale [34]. The test was considered a true maximal test for all the participant as they achieved the criteria established by the American College of Sports Medicine (ACSM) [34]. Key measurements from this test include endurance time, $\dot{V}O_{2}\max$, maximal HR, and peak minute ventilation. Due to the non-linear relationship between oxygen consumption and body mass [35], the $\dot{V}O_{2}$ values were allometrically scaled to prevent errors in metabolic calculations in persons with higher body weight. $\dot{V}O_{2}$ values were raised to a recommended exponent of 0.75 [36].

We built multiple linear regression models to predict absolute $\dot{V}O_{2}\max$ using participants’ characteristics (age, sex, and height) and HR-based features, including the RI and RDI. We included height in all models instead of weight because 1) height had a larger impact on the Ruffier test due to the squatting movement and 2) height had a higher correlation with $\dot{V}O_{2}\max$ in our data. First, we built a regression model separately for RI and RDI to predict $\dot{V}O_{2}\max$, controlling for age, sex, and height. Next, we used a two-step process to select HR-based features for building models. In step 1, we used each of the following features to predict $\dot{V}O_{2}\max$ in a regression model while controlling for age, sex, and height: *P*1, *P*2, *P*3, *P*2 − *P*1, *P*3 − *P*1, *P*2 − *P*3. Each feature’s statistical significance was evaluated using a less conservative p-value (*p* < 0.1). In step 2, we used significant features from step 1, age, sex, and height to build regression models and considered quadratic and cubic polynomials (i.e. *age*^2^, *age*^3^, *height*^2^, and *height*^3^) and also HR-features normalized by these polynomials (e.g. *P*1/*age*^*2*^). The best prediction model was selected based on statistical significance of HR-features, adjusted *r*^2^, and model’s performance metrics sensitivity and specificity.

To evaluate models’ performance in correctly classifying individuals’ CRF levels against ACSM established norms using directly measured $\dot{V}O_{2}\max$, we calculated sensitivity and specificity as follows [32]. We simplified the ACSM classification of fitness levels into 3 categories: poor (combining ACSM’s very poor and poor), fair (ACSM’s fair), and good (combining ACSM’s good, excellent, and superior). Based on the 3 categories, sensitivity was calculated as:

$$Sensitivity=\frac{TP}{TP+FN},$$

where TP was the number of true positive cases, in which the model correctly predicted fitness levels fair and good. FN was the number of false negative cases, in which the model underestimated fitness levels. Specificity was calculated as:

$$Specificity=\frac{TN}{TN+FP},$$

where TN was the number of true negative cases, in which the model correctly predicted fitness level poor. FP was the number of false positive cases, in which the model overestimated fitness levels. Furthermore, we computed the weighted Cohen’s kappa coefficient to assess the agreement between model-predicted $\dot{V}O_{2}\max$ and actual $\dot{V}O_{2}\max$ values using ACSM classifications of CRF.

To evaluate models’ stability, we used: 1) leave-one-out-cross-validation (LOOCV) method and 2) a simulation for calculating and evaluating grant average RMSE. In the LOOCV, we removed one participant from the sample and used data from the remaining participants to build a $\dot{V}O_{2}\max$ prediction model. Data from the removed participant were then used to validate the prediction model. We repeated this process by removing each of the participants. We calculated the standard deviation of the difference between the actual and predicted $\dot{V}O_{2}\max$ values. In the grant average RMSE method, we randomly selected data from 10% of the participants as testing datasets. We then randomly selected participants, from the remaining participants, to form training datasets with increasing sample size, going from 1 to 36. This process was repeated 1000 times. For each size of training datasets, we calculated the grant average RMSE. Lower values of LOOCV and RMSE indicated better accuracy in predicting $\dot{V}O_{2}\max$. We used SAS 9.4 for all statistical analyses (Cary, NC, USA).

## Results

We summarized participants’ characteristics in **Table 1**. The average age of our participants was 31.2 years (SD = 9.9). Participants had a mean BMI of 24.9 (SD = 4.3), with a range of values from 18.6 to 41.2. The $\dot{V}O_{2}\max$ values for these participants ranged from 13.0 to 64.8 ml∙kg^-1^∙min^-1^. The $\dot{V}O_{2}\max$ values for the women participants ranged from 13.0 to 53.0 ml∙kg^-1^∙min^-1^, while those for the men participants ranged from 25.0 to 64.8 ml∙kg^-1^∙min^-1^. These characteristics indicate that this population was a representative sample from the general community, with varying BMI and fitness values.

**Table 1:** Participants’ characteristics.

	Overall N = 40 Mean (SD)	Women n = 22 Mean (SD; Min ~ Max)	Men n = 18 Mean (SD; Min ~ Max)
**Age (years)**	31.2 (9.9)	30.5 (9.7; 20.0 ~ 60.0)	32.0 (10.2; 19.0 ~ 55.0)
**Height (m)**	1.72 (0.10)	1.65 (0.06; 1.57 ~ 1.80)	1.81 (0.07; 1.70 ~ 1.93)
**Weight (Kg)**	74.8 (18.5)	63.9 (12.2; 49.9 ~ 108.9)	88.1 (16.1; 63.5 ~ 121.6)
**BMI**	24.9 (4.3)	23.5 (4.6; 18.6 ~ 41.2)	26.6 (3.3; 20.8 ~ 34.4)
**P1 (beats∙min**^**-1**^**)**	70.2 (10.8)	71.9 (11.6; 49 ~ 98)	68 (9.7; 52 ~ 92)
**P2 (beats∙min**^**-1**^**)**	132.2 (17.1)	135.1 (16.7; 106 ~ 184)	128.7 (17.3; 101 ~ 180)
**P3 (beats∙min**^**-1**^**)**	93.0 (19.6)	93.2 (20.1; 56 ~ 152)	92.6 (19.6; 59 ~ 135)
**RI**	9.5 (4.2)	10.0 (4.3; 4.0 ~ 23.4)	8.9 (4.1; 2.5 ~ 17.1)
**RDI**	10.8 (3.9)	10.8 (3.9; 5.4 ~ 22.2)	10.8 (4.1; 5.5 ~ 18.4)
**V**_**O2max**_ **(L∙min**^**-1**^**)**	2.69 (0.85)	2.13 (0.57; 1.31 ~ 3.24)	3.38 (0.61; 2.23 ~ 4.26)
**V**_**O2max**_ **(ml∙kg**^**-1**^**∙min**^**-1**^**)**	36.7 (10.6)	34.4 (10.3; 13.0 ~ 53.0)	39.6 (10.5, 25.0 ~ 64.8)

(BMI = body mass index; P1 = resting heart rate before squatting; P2 = peak heart rate after squatting; P3 = recovery heart rate 60 seconds after squatting; RI = Ruffier index; RDI = Ruffier-Dickson index.)

### Statistical analysis

We summarized results from three multiple linear regression models for predicting absolute $\dot{V}O_{2}\max$ in Table 2. Models 1 and 2 were based on the RI and RDI, respectively. Model 3 was based on the best-performing HR features developed in this study. Neither RI nor RDI was significant predictor of $\dot{V}O_{2}\max$ (*p* = 0.06 for RI in Model 1 and *p* = 0.32 for RDI in Model 2). However, the developed HR features *P*1 / *height* (*p* = 0.04) and (*P*2 − *P*3) / *age*^*3*^ (*p* = 0.01) were significant predictors of $\dot{V}O_{2}\max$ in Model 3. The adjusted *r*^2^ for the 3 models were 0.587, 0.556, and 0.637, respectively, indicating Model 3 explained the most variability in the $\dot{V}O_{2}\max$ values. The sensitivity (se) and specificity (sp) for the 3 models were se = 0.75, sp = 0.38 for Model 1, se = 0.76, sp = 0.33 for Model 2, and se = 0.79, sp = 0.56 for Model 3. Model 3 had the highest sensitivity and specificity values, indicating it outperformed the other models in correctly classifying ACSM CRF levels. Lastly, Models 1 and 2 had a moderate agreement with the classification of CRF according to the ACSM norms (weighted κ = 0.47 for model 1 and weighted κ = 0.43 for model 2). There was a substantial agreement between model 3 and ACSM CRF levels (weighted κ = 0.60). Overall, model 3 was the best-performing model in predicting $\dot{V}O_{2}\max$ values. Therefore, the final model derived from our analysis is

$$\dot{V}O_{2}\max=3.0143+1.1585\times \mathrm{sex}-0.0268\times (P1/height)+118.7611\times [(P2-P3)/age^{3}],$$

in which sex is coded as 0 for women and 1 for men, and the unit for HR, height and age was beats per minute (bpm), meters (m), and years, respectively.

**Table 2:** Multiple linear regression models to predict VO2 Max.

	B	SE	t	*p*	r	Adj. r^2^	RMSE (L∙min^-1^)	LOOCV (L∙min^-1^)	Sensitivity	Specificity	Kappa
**Model 1**					0.793	0.587	0.552	0.587	0.75	0.38	0.47
*Intercept*	1.45	2.28	0.64	0.53							
*Age*	-0.02	0.01	-2.22	0.03							
*Sex*	1.06	0.28	3.81	< .01							
*Height*	1.04	1.33	0.78	0.44							
*RI*	-0.04	0.02	-1.94	0.06							
**Model 2**					0.775	0.556	0.572	0.610	0.76	0.33	0.43
*Intercept*	0.97	2.36	0.41	0.68							
*Age*	-0.02	0.01	-1.96	0.06							
*Sex*	1.08	0.29	3.73	< .01							
*Height*	1.20	1.38	0.87	0.39							
*RDI*	-0.02	0.02	-1.01	0.32							
**Model 3**					0.816	0.637	0.517	0.543	0.79	0.56	0.60
*Intercept*	3.01	0.60	5.02	< .01							
*Sex*	1.16	0.19	6.21	< .01							
*P*1 /*height*	-0.03	0.01	-2.09	0.04							
(*P*2 − *P*3) /*age*^3^	118.76	44.48	2.67	0.01							

(SE = standard error; RMSE = root-mean-square error; LOOCV = leave-one-out cross validation)

**Fig 1:**
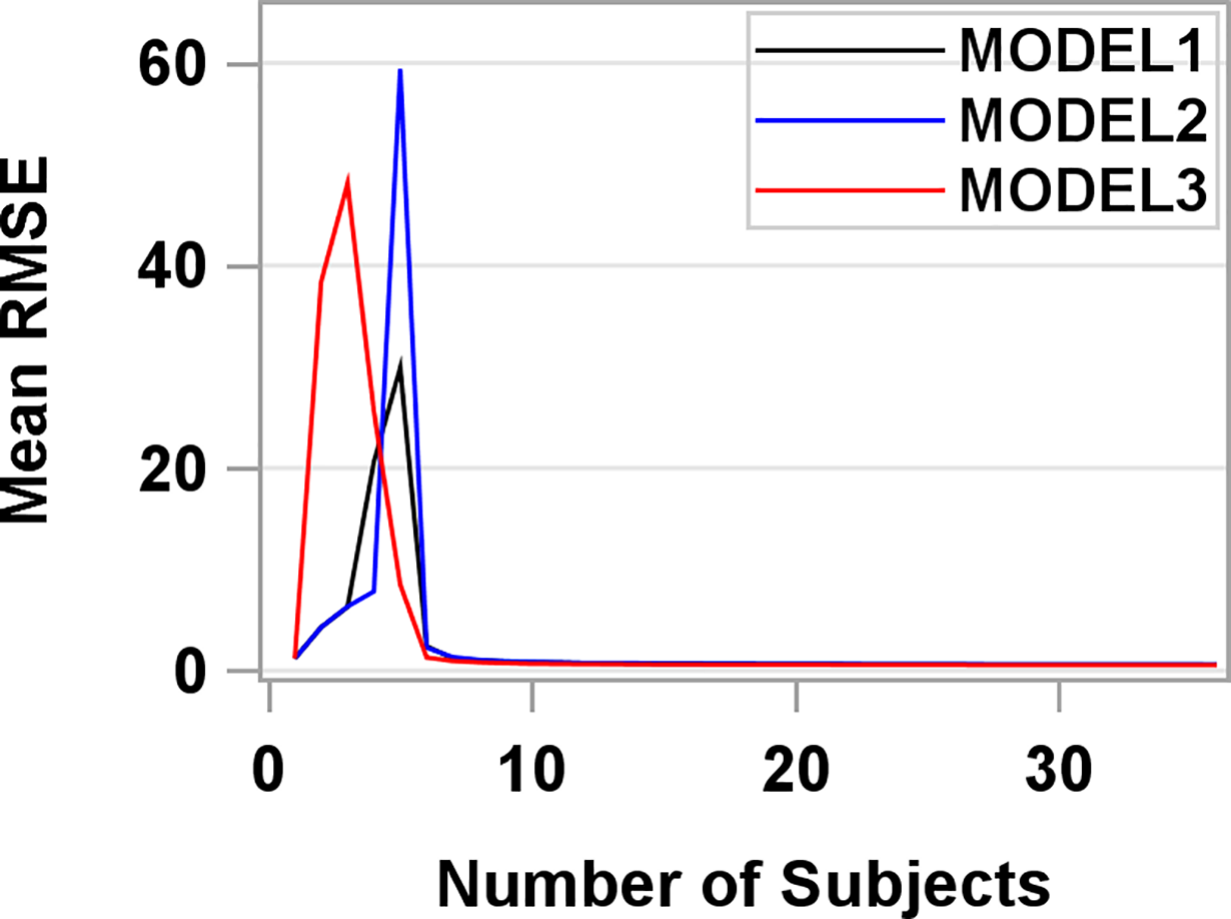
Grand average of the root-mean-squared error (RMSE) by number of participants.

Regarding the models’ stability, the LOOCVs for the three models were 0.587, 0.610, and 0.543 L∙min^-1^, respectively (**Table 2**), indicating that all three models had small prediction errors relative to the $\dot{V}O_{2}\max$ mean (2.69 L∙min^-1^) and Model 3 had the lowest prediction error. We summarized the grand average RMSE for each model in **Fig 1**. As the number of participants included in the training datasets increased, the grand average RMSE decreased and stayed below 0.61 for all three models, indicating that we had sufficient participants in our data to train the 3 models to keep prediction error stably smaller than 0.61 L∙min^-1^ relative to the $\dot{V}O_{2}\max$ mean (2.69 L∙min^-1^).

The fitness assessment sheets based on the $\dot{V}O_{2}\max$ values were provided in **Tables 3 and 4** [37]. For instance, our model shows that a hypothetical 24-year old female patient, with a height of 1.74 meters and weight of 59.0 kilograms, Ruffier HRs values of 69 bpm (P1), 137 bpm (P2), and 94 bpm (P3) has a predicted relative $\dot{V}O_{2}\max$ = (3.0143 + 1.1585 × 0–0.0268 × (69 / 1.74) + 118.7611 × (137–94) / 24^3^) / 59.0 x 1000 = 39 ml/Kg/min. According to **Table 3**, her fitness level is assessed as fair. To facilitate the calculation process, we have created a web application: https://s3.amazonaws.com/vo2/index.html.

**Table 3:** Normative data for VO2 max of women.

Age	Poor	Fair	Good	Excellent	Superior
20–29	< 36	36–39	40–43	44–49	> 49
30–39	< 34	34–36	37–40	41–45	> 45
40–49	< 32	32–34	35–38	39–44	> 44
50–59	< 25	25–28	29–30	31–34	> 34
60–69	< 26	26–28	29–31	32–35	> 35
70–79	< 24	24–26	27–29	30–35	> 35

**Table 4:** Normative data for VO2 max of men.

Age	Poor	Fair	Good	Excellent	Superior
20–29	< 42	42–45	46–50	51–55	> 55
30–39	< 41	41–43	44–47	48–53	> 53
40–49	< 38	38–41	42–45	46–52	> 52
50–59	< 35	35–37	38–42	43–49	> 49
60–69	< 31	31–34	35–38	39–45	> 45
70–79	< 28	28–30	31–35	36–41	> 41

(Values in ml/Kg/min)

## Discussion

In this study, we showed that $\dot{V}O_{2}\max$ can be estimated using key HR features obtained from the Ruffier test. The original RDI and RI scores were not significant predictors of $\dot{V}O_{2}\max$ in our analysis. However, we found that the best $\dot{V}O_{2}\max$ predictors were HR features *P*1/*height* and (*P*2–*P*3)/*age*^3^. Our best-performing model using these two features predicted individuals’ CRF levels with an adjusted *R*^2^ of 0.637, sensitivity of 0.79, and specificity of 0.56. Overall, our analysis provided strong evidence for using the Ruffier test to assess and track CRF for preventive care as a valid alternative to the usual Balke test.

The results showed that the best $\dot{V}O_{2}\max$ prediction model was based on the resting HR (*P*1) and the recovery HR (*P*2 − *P*3) parameters. It has been reported that the resting HR is correlated with $\dot{V}O_{2}\max$ [38]. We found that *P*1 normalized by height (*P*1/*height*) was a better predictor for $\dot{V}O_{2}\max$. Height itself is a strong predictor of maximal aerobic capacity among athletes [39]. Similar fitness prediction equations incorporating body size or height have been used with step tests longer than the Ruffier squat test [40]. For the recovery HR parameter, we found that the difference between the peak HR and 60 second recovery HR was a significant predictor of $\dot{V}O_{2}\max$. In our feature development process, model performance further improved when this feature was normalized by *age*^*3*^. In Sartor et al, the authors found a similar useful feature for predicting $\dot{V}O_{2}\max$, namely the recovery HR linear intercept normalized by *age*^*2*^. However, we re-built the best model in Sartor et al using their original data while only changing *age*^*2*^ to *age*^*3*^ and found that the new model with *age*^*3*^ performed better than their best performing model. This suggests that a recovery HR parameter is needed for predicting $\dot{V}O_{2}\max$, and this parameter may need to be normalized using *age*^*3*^.

There are clear advantages of using a simple squat test compared to the comprehensive treadmill test. The average time required to explain, set up, and complete the Ruffier and Balke tests was 3 and 40 minutes, respectively. The Balke test requires a treadmill, a metabolic cart, and a significant time commitment from the healthcare provider. In contrast, the Ruffier test only requires a metronome and EKG machine which could easily be made available in a clinic. Our results showed that even patients with high BMI were able to perform 30 squats in 45 seconds. Thus, the test could be used for a significant percentage of the primary care patient population. Further, the national average cost for a Balke test is about $3,800, with copays ranging from $200 to 400, whereas the cost of a Ruffier squad test is almost negligible. The Ruffier test provides a valid but inexpensive way to assess and monitor CRF as part of preventive medicine.

Our study has several limitations. First, our sample is a relatively small convenience sample from the general U.S. population. Although we have shown that our sample size is sufficient for the models we built, a large scale epidemiologic study might be needed to further refine model coefficients. Second, participants were healthy individuals. People with conditions such as symptomatic heart disease or moderate-to-severe arthritis would likely not achieve reliable responses. Strengths of the study are the inclusion of men and women with varied BMI values (range 18.6 ~ 41.2), experienced testers, and established $\dot{V}O_{2}\max$ measurement methods. The ability to provide good prediction of CRF across a varied population is critical for the implementation of the squat test for preventive care.

## Conclusions

We evaluated the validity of a 3-minute squat test for estimating CRF in the clinical setting, and built $\dot{V}O_{2}\max$ prediction models using HR features measured from the test. Our best model that included HR features *P*1/*height* and (*P*2–*P*3)/*age*^3^ performed well in predicting $\dot{V}O_{2}\max$ and classifying CRF levels. Our study provides strong support for using the Ruffier test in clinics as an accurate, regular, and inexpensive preventive medicine screening tool to measure and track CRF, and to allow to quantify physical activity as a vital sign.
